# The Effects of Processing Technologies on the Quality of Grain By-Products

**DOI:** 10.3390/foods15183182

**Published:** 2026-09-09

**Authors:** Ruge Cao, Lili Wang

**Affiliations:** 1 College of Food Science and Engineering, Tianjin University of Science and Technology, Tianjin 300457, China; 2 Institute of Food Science and Technology, Chinese Academy of Agricultural Sciences, Ministry of Agriculture, Beijing 100193, China

## 1. Introduction

Global annual cereal output exceeds 2.9 billion tonnes, with wheat, maize, rice, barley, oats, and grain legumes forming the backbone of global food and feed supply chains [1]. Primary grain processing operations—including dry milling, wet fractionation, starch extraction, malting and oil pressing—generate massive volumes of by-products such as bran, rice bran, wheat germ, distillers’ dried grains with solubles, soybean meal, corn fiber and hull fractions, which typically accounting for 15–30% of the raw grain dry weight [2]. The majority of these side streams are currently used as low-grade animal feed or discarded as solid waste, representing a huge loss of inherent bioactive components including arabinoxylans, β-glucan, plant proteins, bound phenolics, vitamins and minerals, while also imposing considerable environmental burdens from waste disposal, greenhouse gas emissions and water consumption.

Against the backdrop of global food security pressure, circular economy transition and growing consumer demand for sustainable plant-based functional ingredients, the high-value valorization of grain by-products has risen to a strategic research frontier. Converting agro-industrial residues into food-grade functional ingredients, bioactive extracts and value-added food matrices can substantially improve resource use efficiency, reduce the environmental footprint of the food chain, and create new economic growth points for the grain processing industry. However, the pathway from underutilized by-product to high-quality food ingredient is technically challenging. Processing technologies directly determine the recovery yield, molecular integrity, functional properties, sensory quality and safety profile of the derived fractions. Systematic elucidation of process–structure–function relationships is therefore essential to unlock the full potential of grain by-products.

This Special Issue entitled “The Effects of Processing Technologies on the Quality of Grain By-Products” was launched to gather cutting-edge research on innovative processing technologies, quality improvement strategies, nutritional enhancement and bioactive component recovery from whole grains and their by-products. In this closing Editorial, we synthesize the current state of the art, identify remaining knowledge gaps, highlight the contributions of the seven published papers, and outline priority directions for future research.

## 2. Advances in Processing Technologies for Grain By-Product Valorization

In recent years, a broad spectrum of physical, biological and non-thermal processing technologies has been developed and applied to modify grain by-products, targeting improved functional properties, enhanced nutrient bioaccessibility and efficient recovery of high-value components.

From a physical modification perspective, conventional thermal treatments are increasingly complemented or replaced by precisely controlled novel physical approaches. Extrusion cooking, with its adjustable shear and temperature and moisture profiles, has been widely used to disrupt fibrous cell wall structures, improve solubility and hydration properties, and inactivate anti-nutritional factors in bran and rice bran [3]. Steam explosion combines high-pressure saturated steam with instantaneous depressurization, effectively loosening lignocellulosic matrices and enhancing the extractability of hemicelluloses and bound phenolics [4]. Superheated steam processing offers a water-free thermal modification route, enabling uniform heat transfer while reducing nutrient leaching compared with conventional wet heating methods [5]. Airflow ultrafine grinding reduces particle size to micron or submicron scale, enlarges specific surface area, and significantly improves water-holding capacity, swelling capacity and sensory fineness of coarse bran fractions [6]. Additionally, surface gelatinization, high-pressure homogenization and pressure-drop processes represent further emerging physical strategies for targeted structural modification of starch and fiber-rich by-products.

Biological processing technologies, including germination, microbial fermentation and enzymatic modification, represent green and mild valorization pathways. Germination activates endogenous hydrolases (amylases, proteases, phytases), degrades anti-nutritional factors such as phytic acid and tannins, and improves protein digestibility and mineral bioavailability [7]. Fermentation with lactic acid bacteria, yeast or filamentous fungi can further modulate flavor profiles, increase soluble dietary fiber content and generate bioactive metabolites [8]. Enzymatic treatments using xylanases, cellulases, proteases and feruloyl esterases enable the highly selective release of bound phenolics, soluble arabinoxylan oligosaccharides and bioactive peptides, with minimal collateral damage to target molecules [9].

Non-thermal processing and rapid detection technologies have also gained increasing attention. Pulsed electric field, ultrasound-assisted extraction, high-pressure processing and cold plasma can permeabilize cell membranes and disrupt cell wall structures at low temperatures, preserving heat-sensitive bioactive compounds while enhancing extraction efficiency [10]. Meanwhile, near-infrared spectroscopy, hyperspectral imaging and other rapid detection methods provide powerful tools for online quality monitoring of by-product streams, supporting precise process control and consistent product quality [11].

## 3. Existing Knowledge Gaps and Technical Bottlenecks

Despite these advances, a core limitation that remains is the lack of mechanistic insight into process–structure–function relationships. Most studies report changes in macroscopic functional properties such as solubility or water-holding capacity, but rarely track the dynamic molecular evolution of arabinoxylans, proteins and starch during processing [12]. This gap hinders targeted process optimization. Compounding this, grain by-products are inherently heterogeneous matrices, with composition strongly influenced by grain variety, growing conditions and primary processing routes. Laboratory-optimized protocols therefore often lack generalizability, and scale-up effects frequently lead to unexpected shifts in product quality and process efficiency.

Additional bottlenecks exist at the quality control and industrial translation levels. Conventional wet-chemical analytical methods are time-consuming and destructive, and robust non-destructive testing solutions adapted to diverse by-product streams remain limited [13]. Furthermore, safety aspects—including processing-induced changes in mycotoxins, anti-nutritional factors and allergenic proteins—are often overlooked in valorization research. At the industrial level, high equipment costs, the absence of dedicated quality standards and low consumer awareness of by-product ingredients all slow the practical adoption of valorization technologies [14].

## 4. Contributions of This Special Issue

This Special Issue compiles seven contributions—six original research papers and one review—covering legume, oat, rice, maize, highland barley and adzuki bean matrices, and addressing several of the aforementioned gaps across biological, physical and non-thermal processing domains.

The review by Wang et al. examines germination as a green biotransformation strategy for improving legume protein digestibility (Contribution 1). It synthesizes two complementary mechanisms: activation of endogenous proteases that modify protein conformation and solubility, and enzymatic degradation of anti-nutritional factors such as phytic acid, tannins and trypsin inhibitors that otherwise reduce protein digestibility. This work provides a mechanistic framework for developing germinated legume ingredients, supporting low-carbon, mild modification of plant protein by-products.

On the quality control front, Li et al. develop a portable near-infrared spectroscopy method for simultaneous quantification of total starch, protein, β-glucan and fat in oat grains (Contribution 2). After comparing eight spectral preprocessing routines and four feature-wavelength selection algorithms, they obtained optimized partial least squares models with prediction R^2^p ranging from 0.768 to 0.903 across the four components. The method enables rapid, non-destructive screening of raw materials and by-product streams, offering a practical tool for on-line process monitoring.

Zhang et al. investigate the moisture-dependent effect of rice protein on starch gelatinization and retrogradation (Contribution 3). Their results show that protein barely affects gelatinization at high moisture but clearly retards retrogradation; at intermediate moisture it effectively inhibits retrogradation, whereas under low-moisture conditions it mitigates water-limited gelatinization but accelerates retrogradation during storage. These findings offer fundamental guidance for incorporating rice processing by-products into starch-based food formulations.

Sukop et al. evaluate steeping optimization combined with pulsed electric field (PEF) pretreatment for gentle recovery of arabinoxylans and proteins from maize wet fractionation streams (Contribution 4). Lactic acid at pH 2.5 maximized arabinoxylan and bound polyphenol retention in the fiber-rich fraction, while hydrochloric acid yielded the highest protein content in the protein-rich fraction. Additional PEF pretreatment further promoted the release of protein, dietary fiber and fat from the fiber matrix and improved functional properties such as water-holding capacity, demonstrating a viable non-thermal intensification route for corn milling by-products.

Li et al. characterize the cooking quality, sensory attributes and volatile profiles of 22 highland barley genotypes from five production regions using gas chromatography–ion mobility spectrometry (GC-IMS) and relative odor activity value analysis (Contribution 5). They identified 44 volatile compounds and screened out varieties with superior texture and flavor performance, linking raw material genotype to end-product quality and supporting variety selection and tailored processing of highland barley and its side streams.

Lee et al. develop a non-thermal pressure-drop pretreatment to enhance adzuki bean rehydration (Contribution 6). Both constant and stepwise pressure-drop modes outperformed conventional thermal soaking, with repeated stepwise treatment further increasing water absorption without thermal damage. Microstructural imaging confirmed localized hilum cracking as the underlying mechanism, expanding the toolkit of mild physical pretreatments for pulse processing.

Finally, Sun et al. propose a CaCl_2_-induced surface gelatinization pretreatment to improve enzymatically prepared porous starch (Contribution 7). The modification increased yield, specific volume, swelling power and oil absorption capacity, and raised fisetin encapsulation efficiency to 93.67%, providing a practical route to convert grain by-product starch into high-performance bioactive delivery carriers.

Taken together, these papers span raw material evaluation, process innovation, functional modification and quality detection, but they do not exhaust the field. Important topics including mycotoxin mitigation, life cycle assessment and consumer acceptance of by-product ingredients remain largely unaddressed and warrant further investigation.

## 5. Perspectives and Conclusions

Looking ahead, priority should be given to developing coupled mild and green processing technologies that enable precise extraction and stabilization of bioactive components from grain by-products while preserving their structural integrity and functional activity. Meanwhile, systematic evaluation of processing impacts on anti-nutritional factors, mycotoxins and allergenic proteins is urgently needed to establish standardized safety protocols for by-product-derived food ingredients.

At the industrial level, pilot-scale validation and economic feasibility analysis should be strengthened to address scale-up challenges. Rapid detection tools such as near-infrared spectroscopy can be integrated with machine learning to enable intelligent process control. Furthermore, life cycle assessment, consumer acceptance studies and dedicated quality standards will be essential to translate technological advances into market-ready solutions and advance the sustainable valorization of grain by-products.

The valorization of grain processing by-products is a multifaceted challenge that requires interdisciplinary collaboration across food science, cereal chemistry, process engineering, nutrition, food safety and sustainability science. The seven papers published in this Special Issue offer novel insights into processing technologies, quality characterization and functional modification of grains and their by-products, representing valuable progress toward a more sustainable grain processing system.

## Data Availability

No new data were created or analyzed in this study. Data sharing is not applicable to this article.
